# Cell-derived extracellular vesicles and membranes for tissue repair

**DOI:** 10.1186/s12951-021-01113-x

**Published:** 2021-11-17

**Authors:** Yuan Ding, Yanjie Li, Zhongquan Sun, Xin Han, Yining Chen, Yao Ge, Zhengwei Mao, Weilin Wang

**Affiliations:** 1 Department of Hepatobiliary and Pancreatic Surgery, The Second Affiliated Hospital, Zhejiang University School of Medicine, Hangzhou, 310009 Zhejiang China; 2 Key Laboratory of Precision Diagnosis and Treatment for Hepatobiliary and Pancreatic Tumor of Zhejiang Province, Hangzhou, 310009 Zhejiang China; 3 Research Center of Diagnosis and Treatment Technology for Hepatocellular Carcinoma of Zhejiang Province, Hangzhou, 310009 Zhejiang China; 4 Clinical Medicine Innovation Center of Precision Diagnosis and Treatment for Hepatobiliary and Pancreatic Disease of Zhejiang University, Hangzhou, 310009 Zhejiang China; 5 Clinical Research Center of Hepatobiliary and Pancreatic Diseases of Zhejiang Province, Hangzhou, 310009 Zhejiang China; 6 Zhejiang University Cancer Center, Hangzhou, 310009 Zhejiang China; 7 MOE Key Laboratory of Macromolecular Synthesis and Functionalization, Department of Polymer Science and Engineering, Zhejiang University, Hangzhou, 310027 Zhejiang China

**Keywords:** Cell membrane, Extracellular vesicles, Cell therapy, Regenerative medicine, Biomaterials, Stem cells

## Abstract

Humans have a limited postinjury regenerative ability. Therefore, cell-derived biomaterials have long been utilized for tissue repair. Cells with multipotent differentiation potential, such as stem cells, have been administered to patients for the treatment of various diseases. Researchers expected that these cells would mediate tissue repair and regeneration through their multipotency. However, increasing evidence has suggested that in most stem cell therapies, the paracrine effect but not cell differentiation or regeneration is the major driving force of tissue repair. Additionally, ethical and safety problems have limited the application of stem cell therapies. Therefore, nonliving cell-derived techniques such as extracellular vesicle (EV) therapy and cell membrane-based therapy to fulfil the unmet demand for tissue repair are important. Nonliving cell-derived biomaterials are safer and more controllable, and their efficacy is easier to enhance through bioengineering approaches. Here, we described the development and evolution from cell therapy to EV therapy and cell membrane-based therapy for tissue repair. Furthermore, the latest advances in nonliving cell-derived therapies empowered by advanced engineering techniques are emphatically reviewed, and their potential and challenges in the future are discussed.

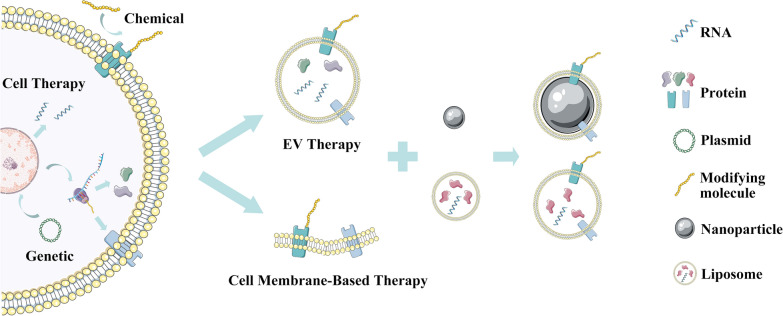

## Introduction

Various diseases and accidents could lead to tissue loss and organ failure. In such situations, cells proliferate and differentiate to regenerate lost cells or tissues, along with the synthesis and resolution of extracellular matrix and subsequent modulation of fibrosis and scar tissue formation. In contrast to nonmammalian vertebrates such as salamanders, in which the capability of scar-free repair and multiple organ regeneration are maintained throughout their life, humans have a limited ability for self-regeneration, which is progressively lost throughout our life. After injury, only a few organs, such as the liver and blood, can completely regenerate in adults, while in most other tissues, fibrosis or scar formation develops as a consequence of the recession of the regenerative capability [1].

Repairing, replacing or regenerating cells, tissues and organs is the key to regenerative medicine to restore impaired function [2]. Cell-derived biomaterials are thus developed, in which viable cells, such as stem cells, progenitor cells, inflammatory cells, cartilage cells, islet cells and liver cells, are injected, grafted or implanted into a patient with haematological [3], cardiovascular [4], and neurological [5, 6] disorders or impaired function of the joint [7], pancreas [8] or liver [9] to effectuate a medicinal effect. Among these methods, stem cells are the most widely used in almost all kinds of diseases due to their alleged therapeutic potency-the ability to differentiate into various cell types to mediate tissue repair and regeneration [10]. However, recently emerging evidence has suggested that the paracrine but not regenerative function of most stem cell therapies predominantly drives the process of tissue repair. In addition, ethical and safety problems have limited the application of stem cells [11]. Together, these new discoveries and challenges emphasize the importance of nonliving cell-derived biomaterials, such as extracellular vesicle (EV) therapy, to fulfil the task of tissue repair.

**Fig. 1:**
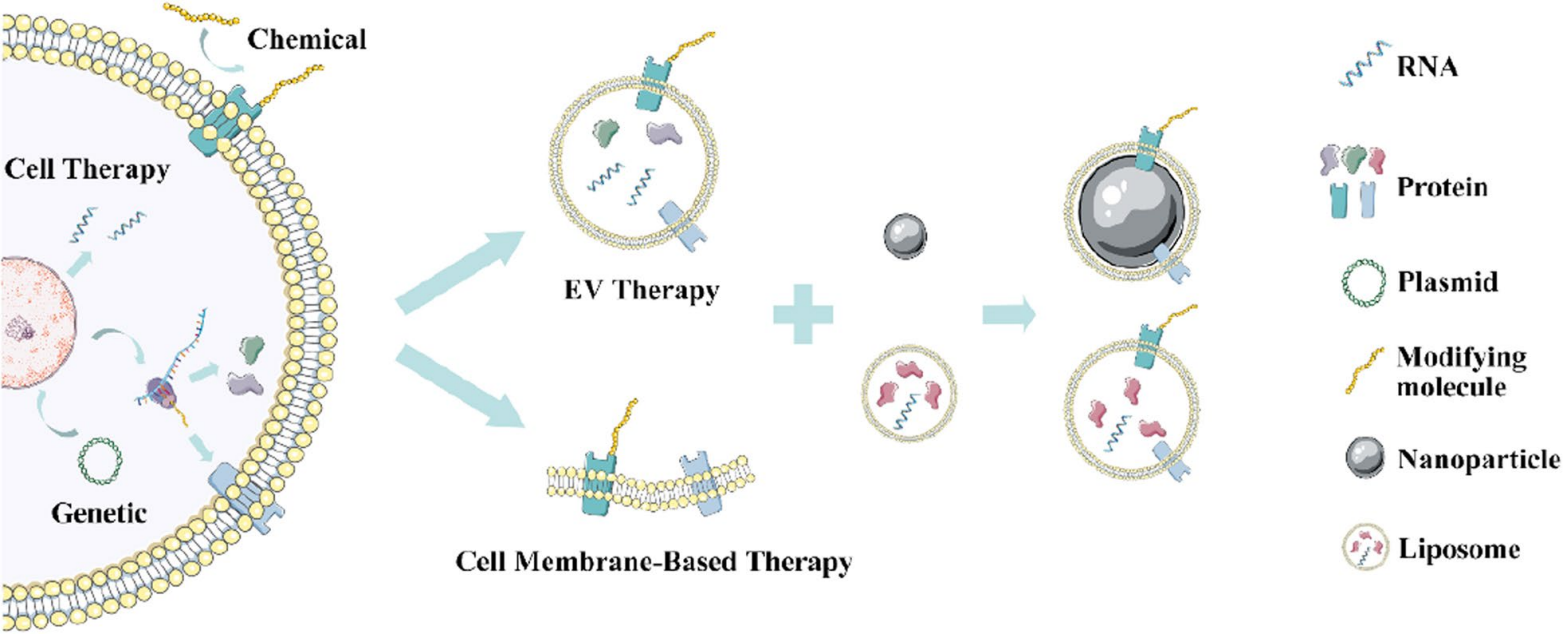
Schematic of cell therapy, extracellular vesicle (EV) therapy and cell membrane-based therapy. All three types of biomaterials utilized in these therapies could be native or engineered. The engineering techniques include genetic approaches and chemical approaches, which could be applied before or after the isolation process of EVs and cell membrane

In comparison with the enduring popularity of research on EV therapy in replacement of cell therapy, the cell membrane technique is a newcomer in the field of regenerative medicine. By virtue of its excellent performance in fighting infection and tumours [12, 13], cell membrane-based therapy has rapidly flourished and extended its influence on tissue repair. In this review, we describe the development and evolution of tissue repair therapies based on cell-derived biomaterials, specifically addressing cell therapy, EV therapy and cell membrane-based therapy (Fig. 1). Since there are already multiple refined reviews illustrating cell therapy and EV therapy in various diseases, after a brief retrospect of these two techniques and the most recent advances in EV therapy, we emphasize the superior capacity of the cell membrane empowered by advanced engineering techniques through a review of studies on cell membrane-based therapy and discuss the potential of cell-derived biomaterials in tissue repair.

## Stem cell therapy

Among various cell therapies, stem cell therapy is one of the best-known and most representative methods applied to various diseases.

### Native stem cell therapy

Two major stem cell types, pluripotent and multi-/unipotent stem cells, are widely studied in the field of stem cell therapy. The former, including embryonic stem cells (ESCs) and induced pluripotent stem cells (iPSCs), can differentiate into all cell types in humans, while the latter only has limited differentiation potential [14].

Haematopoietic stem cells (HSCs), mesenchymal stem cells (MSCs) and stem cells derived from foetal tissues, as multipotent stem cells, were the first to be applied in clinical treatment. Bone marrow transplantations were initially conducted in the 1950s to treat patients with haematological cancers for restoration of a fully functional circulation system through the efficacy of HSCs contained in the marrow [15]. Similar to the first-generation stem cell type, MSCs can be isolated and expanded at a low cost to exploit their properties of trophism, immunomodulation, angiogenic promotion and apoptotic inhibition, thus stimulating many studies and clinical trials [16]. However, research on foetal stem cells is limited to only a few kinds of diseases.

The second generation of stem cell therapies was developed based on the further understanding of the capacity of pluripotent stem cells (PSCs), which are represented by human embryonic stem cells (hESCs) and iPSCs. With broad potency to differentiate into every cell type in the body, PSCs could be indefinitely expanded in vitro [17] for clinical trials covering a broad range of diseases from myocardial infarction (MI) to type 1 diabetes mellitus [18, 19].

Accumulating evidence from the trials above has indicated the favourable translational potential of these techniques. However, their developments have been constrained by ethical and safety concerns, as well as the limited source of therapeutic cells. The use of foetal stem cells is limited not only by controversy and ethical concerns regarding the procurement of foetal tissue but also by the difficulty of isolating a sufficient number of stem cells from every foetal donor with stemness loss during large-scale culture in vitro. Such a situation has made clinical translation of therapies relying on foetal stem cells difficult [20]. Moreover, the direct use of PSCs might have a risk of tumour formation or tissue heterotopia in vivo. Ethical concerns regarding the unavoidable procedure of human embryo destruction have hindered the application of hESCs. Additionally, safety issues for the potential genetic instability of hESCs and iPSCs are a challenge [18].

Therefore, among these therapies, the use of MSCs in regenerative medicine has several potential advantages: fewer ethical and safety concerns and more available sources, including umbilical cord tissue, fat tissue, central nervous system tissue and bone marrow. MSCs are capable of differentiating into multiple cell types, such as osteoblasts, chondrocytes, and myocytes, to remodel bone, cartilage and vascular tissue [1]. Unfortunately, MSCs have been under harsh criticism, as several clinical trials based on these cells have failed to show functional recovery [21, 22]. Studies have indicated that these cells rapidly disappear from the target location after systemic or direct injection [23]. Even though recovery indeed occurred at the damage site, the effects often resulted from the paracrine effect of the implanted cells rather than their regenerative capacity [21]. However, despite the inadequate recognition of MSC functions, efforts are frequently being made to utilize these cells for clinical treatment, even with the highly controversial use of uncharacterized cells [24]. To date, no MSC-derived therapies have been approved by the US Food and Drug Administration.

### Engineered stem cell therapy

**Fig. 2:**
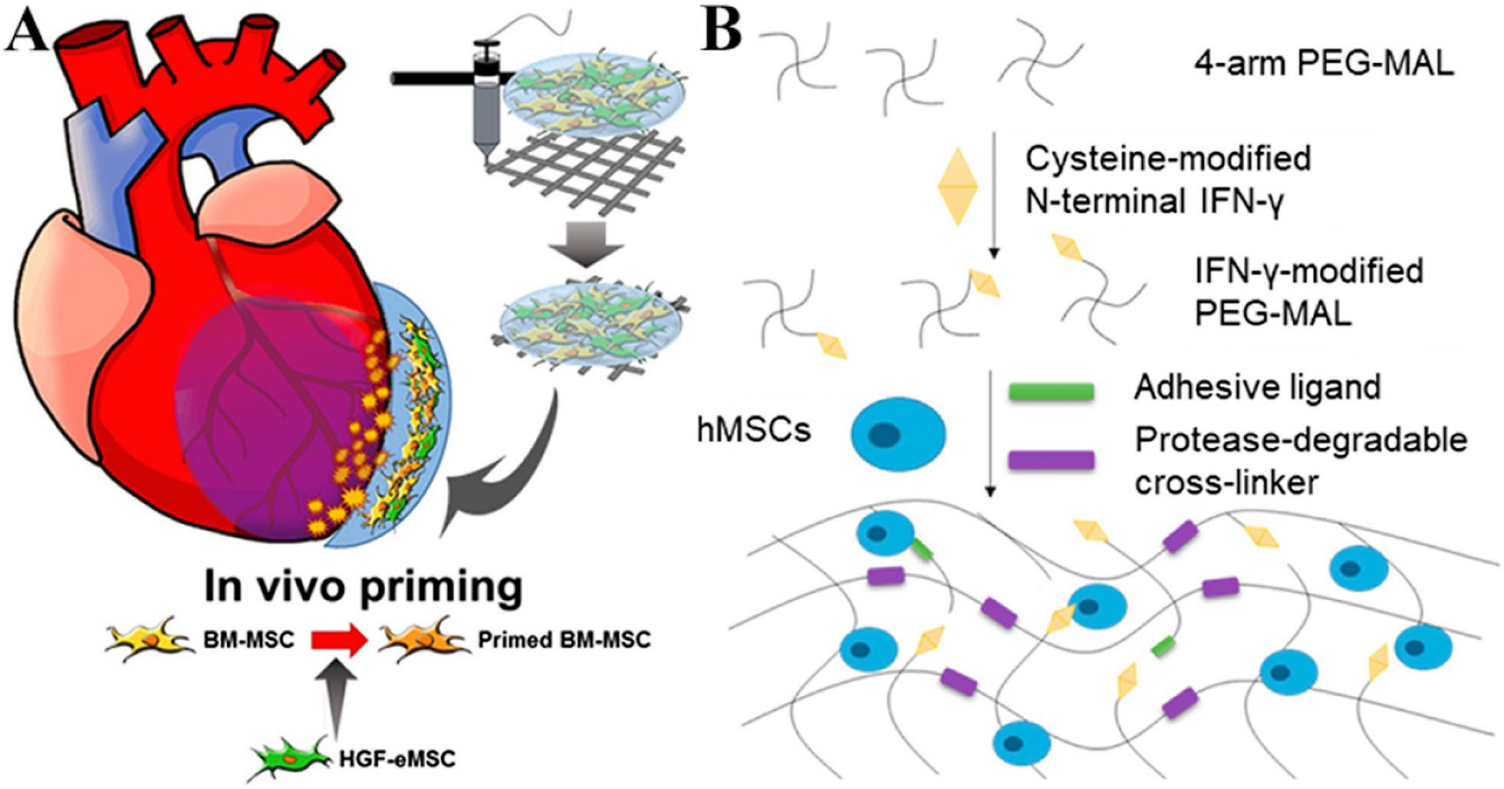
Engineered stem cell therapy. **A** Scheme illustrating an example of genetic engineering of stem cells. Bone marrow–derived mesenchymal stem cells (BM-MSCs) were primed by transduction-mediated hepatocyte growth factor–expressing MSCs (HGF-eMSCs) for enhanced efficacy of vascular regeneration and cardiac function restoration in myocardial infarction models. Reproduced from [25]. © The Authors, some rights reserved; exclusive licensee AAAS. **B** Concept of hydrogels tethering interferon-gamma (IFN-γ) and encapsulating MSCs. Reproduced from [26], copyright 2019, with permission from Elsevier

Given that the functional improvements observed in stem cell therapies might result from paracrine actions, the concept of regenerative medicine has been gradually extended from the original concept of regeneration to replace injured tissues with cell-dependent, nonspecific effects covering trophism or immunomodulation [20]. Consistent with that, the focus of this field has migrated from direct tissue regeneration by stem cells to tissue repair orchestrated by their paracrine effects. Therefore, in recent studies, engineering approaches have been applied to add specific targeting and paracrine factors or drugs to improve the efficacy of stem cells and/or broaden the range of their application. In those situations, stem cells serve as delivery vehicles for exogenous proteins or drugs or overexpressed endogenous agents [11] (Fig. 2A).

Exogenous proteins can be introduced into cells by exogenous gene expression via virus-mediated transduction, and the cellular production of endogenous proteins eliciting reparative effects (e.g., growth factors) could be enhanced through similar approaches. However, risks are associated with commonly used RNA-based retroviruses, as they might promote proto-oncogene expression or disrupt the function of tumour suppressor genes by integrating into their sequences, which leads to insertional mutagenesis considering the longevity of stem cells in vivo*.* After receiving gene therapy against X-linked severe combined immunodeficiency via transplantation of retrovirus-transduced CD34^+^ haematopoietic cells, four out of nine patients developed leukaemia within 31–68 months [27]. Self-inactivating lentiviruses containing deletions in their 3′ long terminal repeat region might reduce the mutagenic possibility but add complexity and cost to drug development [28]. The utilization of other tools, such as the CRISPR–Cas system, that enable more efficient design of new therapeutic elements [29] is challenged by their high frequency of off-target editing [30]. However, the efficiency and convenience provided by CRISPR–Cas systems still make them the most popular gene editing tool for the establishment of engineered stem cells [31].

Other engineering approaches have recently been developed to enhance the efficacy of stem cell therapy. For example, the application of hydrogel-tethered interferon-gamma (IFN-γ) could increase the immunomodulatory functions of encapsulated MSCs and avoid the need for ex vivo manipulation. These MSCs could halt T-cell proliferation and dendritic cell differentiation, accelerating healing of colonic mucosal injuries [26] (Fig. 2B).

However, a phase IIb trial of brain damage treated with modified MSCs transfected with a Notch intracellular domain revealed no statistically significant enhancement of endogenous NSC differentiation [32]. In addition to unsatisfactory clinical effects, stem cell therapy faces more challenges in manufacturing and medical safety. The complicated and sophisticated engineering procedures of therapeutic cell production bring barriers to manufacturing. For prevention of an inflammatory response, personalized engineered stem cells are required for each patient, adding costs to the manufacture of stem cells for clinical application [11].

The risk of pulmonary embolism and tumorigenesis needs to be defined [33], as evidence suggests that the angiogenic and immunosuppressive natures of MSCs might promote tumour growth [34]. Once PSCs are transplanted into patients, the immune-evasive property endowed by their universal compatibility might cause difficulty in controlling their behaviour in vivo [35]. Taken together, these challenges slow down the translation of stem cell therapy in clinical applications.

### Other cell therapies

In addition to stem cells with cell survival promotion, immunomodulatory and angiogenic capacities, some other cells with similar or more regenerative abilities are adopted in tissue repair. For example, hepatocytes can rapidly enter the cell cycle when injuries occur in the liver and thus exert a better regenerative effect on liver failure. Because of the lack of donors, hepatocyte transplantation has been used intensively in the bridging process of orthotopic liver transplantation. However, these therapeutic cells might be transplanted to a critical environment for survival and expansion. Macrophages activated by cellular necrosis and apoptosis might induce hepatocyte replicative senescence, which impairs the therapeutic effect of hepatocytes [9].

For the reasons mentioned above, the inflammatory response modulation of tissue repair has been increasingly studied. When injury occurs, the inflammatory response is activated by the local release of chemokines by innate immune cells recruited to the injury site, which contributes to the tissue repair response. The degree and duration of the response determine the final outcome. If the proinflammatory response does not fade away in a timely manner, the homeostasis of the injured tissue would not be restored, and tissue function would be further debilitated [36]. The therapeutic potential of macrophages [37] and a unique immunosuppressive subset of regulatory T cells (Tregs) [38, 39] has thus been explored to enhance the activity of tissue repair in various preclinical models of inflammatory diseases. However, for patients with abrupt injuries such as stroke or traumatic brain injury, it is difficult to apply autologous cells in a timely manner because it takes time for vitro expansion of a sufficient number of therapeutic cells [6].

## Extracellular vesicle therapy

Since the large-scale application of cell therapy still faces numerous barriers, researchers are trying to search for novel approaches to achieve tissue repair. EV therapy appeared at the right time, as it is safer, controllable by eliminating the disadvantages of living cells, and more convenient for efficacy enhancement by bioengineering techniques [40].

### Characteristics of EVs

As mentioned above, cell therapies mainly exert their therapeutic effects via secretion of paracrine factors. In addition to common secretory pathways, proteins and RNAs can be secreted and delivered as vesicles generated from the plasma membrane, which are called extracellular vesicles. EVs play important roles in promoting in vivo tissue repair by maintaining tissue homeostasis and modulating several physiological pathways through intercellular communication. As a repair component of the stem cell secretome, EVs have potential as a therapeutic tool in the field of regenerative medicine [41]. Therefore, interest has grown in cell-free therapies to replace the role of transplanted cells in some fields, leading to a better defined and low-cost product and avoiding ethical and safety issues.

Based on size, density, manner of release from cells, etc., EVs can be divided into three subgroups: exosomes, microvesicles, and apoptotic bodies [42]. Although distinct in biogenesis, isolation of a typical kind of EV from their heterogeneous population could be unavailable, as their physiochemical characteristics and biological markers overlap [43]. The term EVs is widely used to comprise all types of these vesicles.

Structurally, EVs exert intercellular communication through their membrane and cargos in the lumen. The EV membrane maintains features from its original cell membrane and thus could mediate the interactions between EVs and target cells [44]. With their cell-like membranes and various proteins and RNAs in the lumen, EVs can activate target cells through intracellular cargo delivery or direct activation of receptors on their plasma membrane [45]. After endocytosis, EVs are either trafficked to lysosomes by endosomes for degradation or fused with the membrane of endosomes or the plasma membrane, releasing their luminal cargoes [46].

### Native EV therapy

Studies on the tissue repair potential of EVs can be traced back to 2009, when EVs secreted by MSCs were expected to exert major therapeutic effects in a kidney injury model [47]. Therefore, more studies have revealed the repair-promoting effects of EVs isolated from MSCs in many tissues, including cardiovascular [40], neurological [48], musculoskeletal [49] and pulmonary indications [50]. The tissue repair mechanisms of MSC‐derived EVs are mediated by immune modulation [51], angiogenic enhancement [52], apoptotic inhibition [53], and reduction of fibrosis [54]. Recent studies further revealed novel fields for stem cell-derived EV application in tissue repair. Sun et al. found that human umbilical cord mesenchymal stem cell (hucMSC) EVs reduced blood glucose levels by partially relieving β-cell destruction and reversing insulin resistance in type 2 diabetes [55]. Choi et al. found that EVs from adipose-derived stem cells (ASCs) could provide photodamaged dermal fibroblasts with UV protection, collagen biosynthesis, DNA repair and enhanced cell migration [56]. Inhalation of EVs from lung spheroid cells, which are isolated from three‐dimensional multicellular lung spheroids containing a natural mixture of lung stem cells and supporting cells, was also found to restore the organized alveolar structure and decrease both collagen accumulation and myofibroblast proliferation, which alleviated injuries and fibrosis in different models of lung diseases [57].

In addition to EVs derived from stem cells, EVs from other cells are employed in the repair process [58], inheriting the tissue repair capacities of their original cells, such as inflammatory cells [59, 60]. Among them, M2 macrophage EV‐guided cell reprogramming was established to directly convert M1 macrophages to M2 macrophages to accelerate wound healing by enhancing angiogenesis, re‐epithelialization, and collagen deposition [61]. In addition, Hu et al. discovered that EVs from dermal papilla spheroids could promote the development of hair follicles by effectively accelerating the cycle from telogen to anagen [62].

Xenogenic cell-derived EVs are also surprisingly able to induce tissue repair. EVs from newt limb explant cells (A1 cells) were similar to mammalian EVs in diameter, structure, and proteins from the lumen and membrane surface. Nevertheless, both RNAs and proteins contained in A1-EVs showed significantly higher levels per vesicle. Numerous unique RNAs and proteins were found in these EVs, many of which were elements expressing nuclear receptors, membrane ligands and transcription factors. After the administration of these A1-EVs to mammalian cardiomyocytes, increased gene expression of survival promoting the PI3K/AKT pathway was detected, which prevented the oxidative stress-induced apoptosis of cardiomyocytes [63].

Compared to cell therapy, EVs have abundant advantages as they are nonliving entities. EVs could provide similar therapeutic effects as cell therapy and also overcome their limitation of poor survival after implantation, as they are directly internalized by the target cells [40]. In addition, EVs from cells such as MSCs could ameliorate some adverse effects related to MSC therapy, including adverse immune responses, lung vessel obstruction, and malignant transformation [48]. Furthermore, the clinical translation of cell therapy is hampered by limited sources and high manufacturing costs; in contrast, EVs require simplified frozen storage and thawing conditions for their extended stability, which potentially allows them to retain functionality after being lyophilized for a ready-made product, reducing the large-scale production costs compared to those of cell therapy [64].

However, there are still aspects of EV therapy that have yet to be improved. EVs are quickly cleared in the liver, lungs, and spleen with a short half-life in minutes after systemic administration. Rapid clearance of EVs from the injection site also indicates that very limited improvement could be achieved through local administration [65], which limits their clinical application. However, the average EV yield from 10^6^ cells is 1 to 4 μg/day, which could barely meet the demand for a dosage of 100 to 500 μg per patient of EVs in a clinical trial [66].

### Engineered EV therapy

Only a small amount of EVs can reach the target site and exert therapeutic efficacy, which impairs the effects of EV therapy and raises the required yield of EVs [67]. Therefore, methods have been proposed to improve the bioavailability and efficacy of EVs [68]: (a) delivering EVs to specific tissues or cell types by modifying targeting molecules and/or exogenous guidance; (b) enhancing the therapeutic factors contained in the EVs; (c) increasing the intracellular release of EV cargo into the cell cytoplasm; and (d) controlling the spatial and temporal release of EVs for a more local and sustained delivery of EVs.

#### Engineered EVs for targeting

Targeting EVs to specific tissues and organs could be improved by loading targeting molecules in the lumen or on the membrane of EVs. The molecules modified on the membrane could interact with specific receptors on target cells or components in the microenvironment surrounding the target [69]. For example, the cyclo(RGDyK) peptide could target ischaemic regions in the brain [70], and rabies viral glycoprotein (RVG) could guide EVs towards neurons [71]. Similarly, peptides screened via phage display biopanning could guide EVs to the ischaemic myocardium [72] and cardiomyocytes [73].

**Fig. 3:**
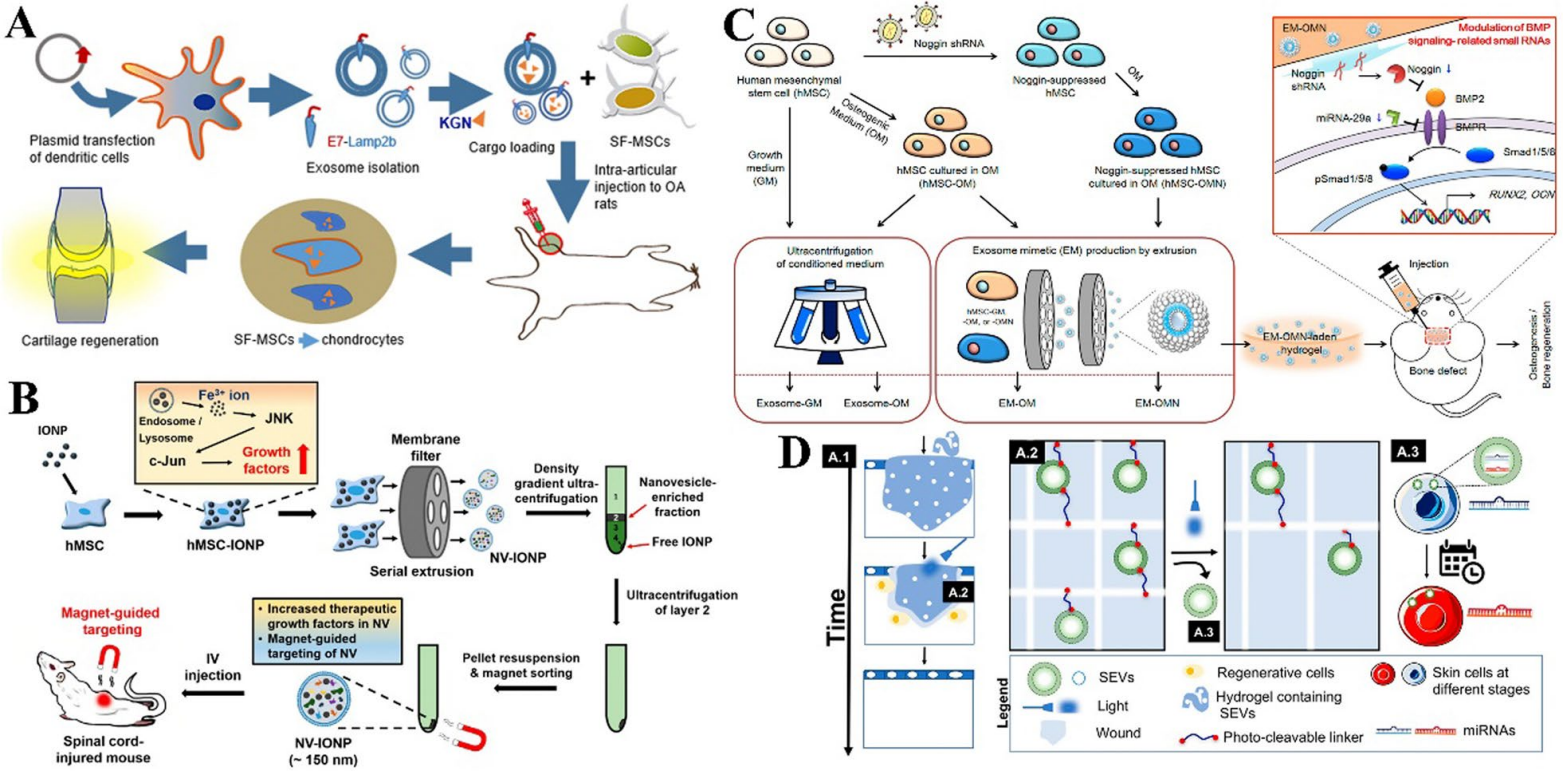
Engineered extracellular vesicle (EV) therapy. **A** Schematic illustration of genetical modification on the surface of dendritic cell-derived EVs with mesenchymal stem cell (MSC)-binding peptide E7. Reproduced from [75], copyright 2020, with permission from Elsevier. **B** Schematic illustration of iron oxide nanoparticle (IONP)–incorporated EVs extruded from IONP-treated MSCs being guided to the injured spinal cord under exogenous magnetic guidance. Reproduced with permission from [76]. Copyright 2018 American Chemical Society. **C** Schematic illustration of EVs from MSCs downregulated expression of a natural bone morphogenetic protein antagonist, exerting improved osteogenic ability. Reproduced with permission from [77]. Copyright 2020 American Chemical Society. **D** Schematic illustration of a coordinated release of small EVs (SEVs) via remotely triggerable hydrogels, which could be cleaved by exposure to blue light at intervals altered on demand. Reproduced with permission from [78]. Copyright 2019 American Chemical Society. SF-MSCs, synovial fluid-derived MSCs; OA, osteoarthritis; hMSC, human MSC; NV, nanovesicle; SEVs, small EVs

Apart from physical [72] or chemical [70, 74] incorporation of targeting molecules into the membrane mentioned above, genetic modification has also been used to modify the surface of EVs with the expression of specific peptides. For example, Xu et al. elevated the targeted drug delivery capacity of dendritic cell (DC)-derived EVs to MSCs by plasmid transfection, which induced overexpression of MSC-binding peptide E7 fused with the EV membrane protein, which strongly promoted chondrogenesis of MSCs [75] (Fig. 3A).

In addition to membrane modification, exogenous guidance could lead to targeted delivery of EVs to action sites. For instance, Kim et al. fabricated iron oxide nanoparticle (IONP)-incorporated EVs from IONP-treated MSCs to enhance the targeting ability of EVs, in which IONPs acted as a magnet-guided navigation tool, significantly increasing the EV accumulation in the injured spinal cord under exogenous magnetic guidance [76] (Fig. 3B).

#### Methods to optimize the yield of EVs

The yields of EVs and therapeutic cargos in EVs could be enhanced by modulation of EV-secreting cells by culturing them in stress-inducing conditions [79–81] or transfecting them with exogenous compounds [77, 82].

Referring to the former approach, Li et al. applied cytochalasin B in macrophages to relax the interaction between the cytoskeleton and membrane and thus to stimulate macrophage-derived EV secretion. This approach was relatively convenient and had a high efficiency: approximately 0.3 mg of EVs could be isolated from 10^7^ macrophages, while the same number of macrophages in the control group only produced 0.06 mg of EVs [80]. Yang et al. used a 3D graphene scaffold as the matrix for hucMSC culture to obtain EVs. In contrast to EVs obtained from 2D culture, EVs from 3D culture had distinct levels of miRNAs and proteins. Mediated by these unique EV cargos, the expression of α‐secretase was upregulated and that of β‐secretase was downregulated in target cells to reduce Aβ production, which ameliorated the cognitive and memory recession in mice with Alzheimer’s disease (AD), demonstrating enhanced therapeutic potential [81].

Regarding the transfection method, Ran et al. demonstrated that via transduction of myostatin propeptide with its inhibitory domain fused into the second extracellular loop of CD63, the propeptide could be overexpressed and anchored to the EV membrane, which significantly enhanced the efficacy of EVs on myostatin inhibition, accelerating muscle regeneration and growth [82]. In contrast to generation of therapeutic EVs by upregulating gene expression, Fan et al. genetically modified MSCs to downregulate their expression of noggin, a natural bone morphogenetic protein antagonist, from which the isolated EVs could preferably exert their osteogenic ability [77] (Fig. 3C).

Either through culturing cells under IFN-γ stimulation or through genetic cell engineering, Su et al. obtained a high concentration of PD-L1 in EVs, which suppressed T cell activation after specific binding to PD-1 on the cell surface, significantly speeding up wound contraction and re-epithelialization during the inflammation phase [83].

Another novel technique has been developed to enhance EV cargo in recent years [76, 77, 84]. Jeong et al. generated EVs mimicking nanovesicles by extruding embryonic stem cells through microfilters. As the enclosed lipid bilayer and cellular contents were well preserved in these nanovesicles, they could exert therapeutic effects in primary murine skin fibroblasts by upregulating the expression levels of beneficial mRNAs and proteins in fibroblasts and enhancing their proliferation rate and number, potentially contributing to tissue recovery or wound healing processes [84].

#### Engineered EVs for intracellular trafficking

The present understanding of the mechanism of intracellular EV cargo release is limited. As a large proportion of EVs degrade in the endolysosomal system after internalization, recent studies have aimed to modulate the cell endocytic capacity and surface modifications of EVs to optimize intracellular trafficking and enhance endolysosomal escape [85]. Strategies have been proposed to enhance intracellular cargo release. For enhanced endolysosomal escape, the endolysosomal membrane could be disrupted by the coating combination of cationic lipids or/and pH-sensitive fusogenic peptides on EVs, which led to the efficient cytosolic release of EV cargos. For enhanced internalization, cell-penetrating peptides such as human immunodeficiency virus transactivator protein (TAT) and/or penetratin could be utilized to coat EVs, inducing active micropinocytosis for cargo release. Other stimuli-sensitive nanoparticle (NP)-based drug delivery systems are widely used in the responsive release of therapeutic elements [86].

Recently, it was reported that in an acidification-dependent manner, a proportion of internalized EVs could integrate with the limiting membrane of endosomes and lysosomes, leading to the exposure of their cargos to the cytoplasm, which might inspire new approaches [87].

#### Engineered EVs for controlled release

EV uptake by target cells could be enhanced by locally administering EVs at the site of injury, while slowing the clearance of EVs from the administration site is also critical. Thus, several strategies have been established to achieve sustained release of EVs at the injured site to enhance EV presentation to recipient cells by utilizing multiple biomaterials, including hydrogels incorporating hyaluronic acid [88], alginate [89] and even 3D‐printed titanium alloy scaffolds [90].

Recent progress has been made in the spatial and temporal release of EVs. Silva et al. applied allogenic EVs from ASCs with a thermoresponsive gel to a porcine fistula model. After gelling at body temperature, the gel retained EVs in the entire fistula tract, completing 100% fistula healing [91]. Notably, the therapeutic effects of EVs in hydrogels seem to rely on the kinetics of the release process. Antunes et al. showed that via remotely triggerable hydrogels that were cleaved by exposure to blue light, a coordinated release of EVs could be achieved during the skin regenerative process. Through exposure to blue light at intervals altered according to the healing rate of the wound, the hydrogel was triggered to gradually degrade to release EVs in a controlled manner [78] (Fig. 3D).

## Cell membrane-based therapy

EV therapies have various advantages in tissue repair and regenerative medicine endowed by the functions of the EV membrane and cargos in its lumen, which mimic the capacities of their original cells to exert intercellular communication. However, there is still a long way to go for the clinical translation of EV therapies, considering the complexity of EV isolation and the uncertainty of various nontherapeutic factors in EVs. On the one hand, there is no consensus on the optimal approach for EV isolation, as each isolation technique has its own advantages and limitations [92]. On the other hand, EV cargos could lose therapeutic efficacy in the process of engineering [93], and various bioactive factors in EVs might cause unpredictable side effects [46]. These factors limit the manufacture and efficacy of EVs and might lead to safety concerns about their unexpected adverse reactions. Cell membrane technology, as a more engineering-dependent approach that combines the function of the membrane and defined therapeutic elements, has been introduced in the field of regenerative medicine.

### The development of cell membrane technology

Cell membrane coating technology was first reported in 2011. By transferring the outermost layer of a cell directly onto the surface of an NP, Zhang’s group established this technique, which utilized entire cell membranes as the NP coating material. This technique could faithfully preserve the complexity of the membrane, including all of its lipids, proteins, and carbohydrates, which enable the membrane‐coated NP to exhibit as many properties as the source cell. With this technique, red blood cells (RBCs) became the first source of membrane material [94]. RBCs are known for their relatively long lifetime of up to 120 days in humans, which is mainly mediated by their surface marker CD47, and thus possess the ability to circulate for extended periods of time for the delivery of the cargo they carry [95]. The NPs coated by the RBC membranes had a similar extended lifespan in a mouse model after intravenous administration [94], which suggested the potential of the utilization of cell membrane coatings with therapeutic elements to treat diseases.

Since then, membranes from multiple types of cells, including RBCs, platelets (PLTs) [96], leukocytes [97] and tumour cells [13], have been utilized in NP coatings and applied for disease treatment. For example, Hu et al. constructed RBC membrane-coated nanosponges with an absorptive capacity to divert membrane-disrupting toxins away from their cellular targets, which decreased the damage induced by staphylococcal alpha-haemolysin in a mouse model [12]. Their group also took advantage of the immunomodulatory and adhesive capability associated with PLTs and enclosed polymeric NPs in the PLT membrane to evade cellular uptake by phagocytes and avoid complement activation usually induced by nude particles. The adhesive properties of PLTs to damaged vasculatures and pathogens endowed cloaked NPs with the ability to selectively target injured sites and enhance binding to bacteria in animal models of coronary restenosis and systemic bacterial infection, enhancing therapeutic efficacy [96]. Parodi et al. showed that apart from processing the immune escape ability, leukocyte membrane-coated nanoporous silicon particles could release the payload in the target site after transport across the inflamed endothelium through receptor–ligand interactions between membranes, showing enhanced accumulation in a tumour [97].

### Native cell membrane-based therapy in tissue repair

In the past, cell membrane coating technology was mainly utilized in the fields of infection and tumour treatment. Recently, cell membrane-based therapy has extended its application to tissue repair with a variety of cell membranes and bioengineering technologies employed.

#### Stem cell-mimetic cell membrane-based therapy

The use of cell membrane technology in tissue repair was previously explored to solve the problems in stem cell and stem cell-derived EV therapy. As an alternative approach to EVs, packaged secreted factors from MSCs were coated with stem cell membranes [98, 99]. These stem cell-like microparticles (MPs) exhibited similar surface antigens and secretomes to those of genuine stem cells. They could promote cardiomyocyte functions and showed cryopreservation and stability with lyophilization and could also avoid the tumorigenicity and immunogenicity risks of living stem cell transplantation [100]. Direct injection of these MPs in an animal model of acute MI could promote angiogenesis and alleviate ventricle remodelling.

Similarly, in addition to stem cells, RBC-coated NPs that carry beneficial regenerative factors from MSCs can be applied to repair tissue. In one situation, RBC membrane-coated NPs achieved increased blood stability with lower internalization by macrophages and were able to remain in the liver, where the NPs could promote liver cell proliferation and ultimately mitigate carbon tetrachloride-induced liver failure in a mouse model [101].

**Fig. 4:**
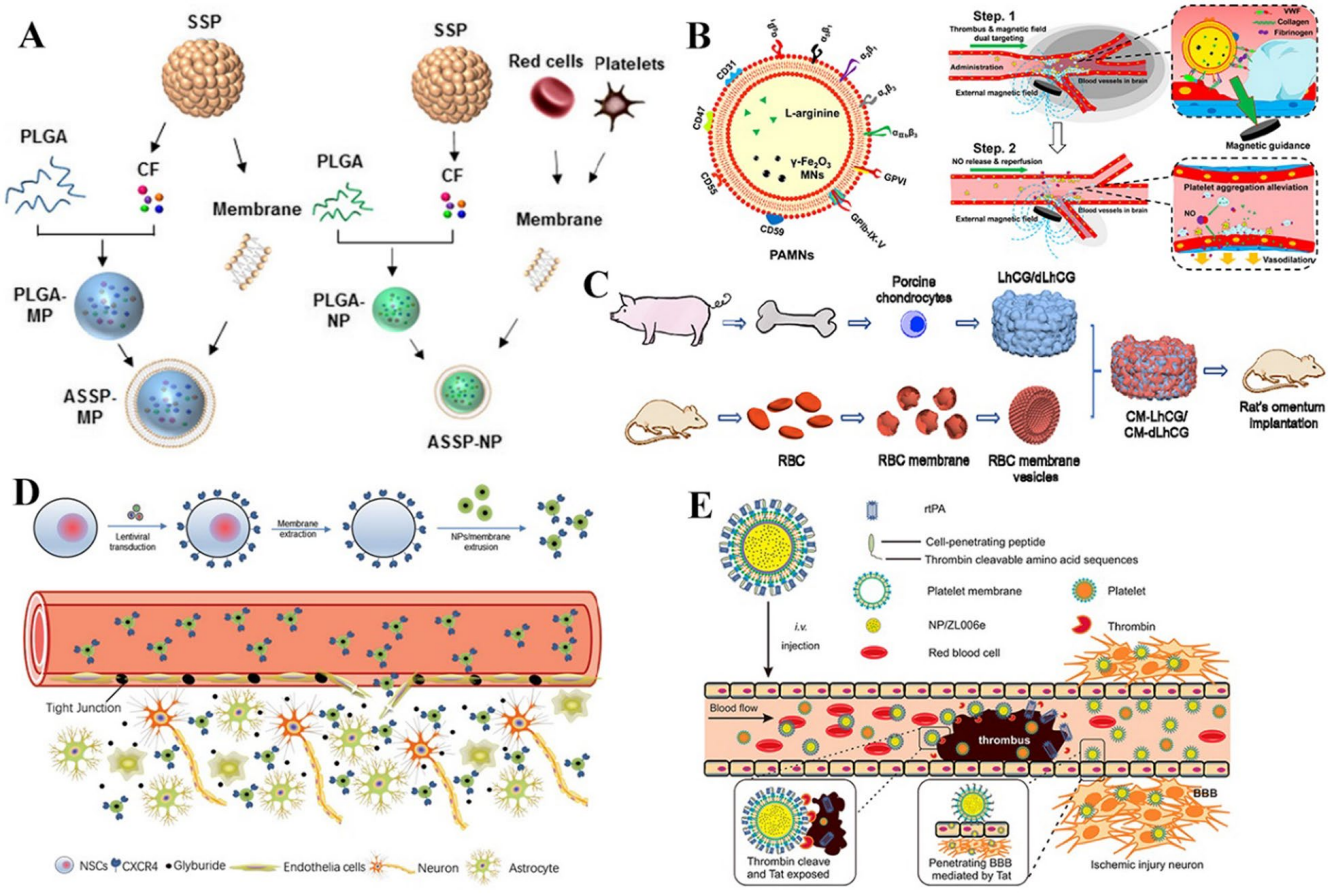
Native cell membrane-based therapy and therapy with genetic or chemical engineering of cell membrane. **A** Schematic illustration of the size-variable artificial stem cell spheroid (ASSP), combining the paracrine functions of three-dimensional (3D) SSPs and different types of coating membranes. Reproduced from [102]. © The Authors, some rights reserved; exclusive licensee AAAS. **B** Schematic illustration of promoted targeting capacity achieved under the navigation of an external magnetic field for cell membrane coating γ-Fe2O3 magnetic NPs. Reproduced with permission from [105]. Copyright 2020 American Chemical Society. **C** Schematic illustration of xenogeneic cartilage tissue graft coated by autologous red blood cell (RBC) membrane of high stability, inducing less inflammatory responses. Reproduced from [106], Copyright 2020, with permission from Elsevier. **D** Schematic illustration of CXCR4-overexpressing neural stem cell membranes with enhanced efficacy in stroke treatment with poly(lactic‐co‐glycolic) acid nanoparticles (PLGA NPs) loaded with anti‐oedema agents [107]. Reproduced with permission from [105]. Copyright 2019, Wiley. **E** Schematic illustration of platelet membrane-coated NPs conjugated with TAT and rtPA that could be sequentially functioned after the responsive cleaving of their connecting structure. Reproduced with permission from [108]. Copyright 2019 American Chemical Society. MP, microparticle; CF, cocktail factor; PAMNs, platelet membrane envelope loaded with l-arginine and γ- Fe2O3 magnetic nanoparticles; CM, cell membrane; LhCG, living hyaline cartilage graft; dLhCG, decellularized LhCG; BBB, blood–brain barrier

Realizing that paracrine factors are the key therapeutic element in stem cell-mimetic therapy, Zhang et al. introduced a size-variable artificial stem cell spheroid (ASSP) technique, combining the paracrine functions of three-dimensional (3D) SSPs for vasculature regeneration. First, for generation of a hypoxic microenvironment for enhanced proangiogenic factor secretion, MSCs were induced to aggregate into 3D spheroids. Then, depending on the disease and corresponding action site, SSP-secreted factors were integrated into different types of membranes selected to exert their function, forming MPs/NPs with MSC/RBC-PLT hybrid membrane–derived surface coatings. By virtue of the easily controllable size of the ASSP particles, the local administration of ASSP MPs and systemic delivery of ASSP NPs could provide superior revascularization effects in the ischaemic tissues of hindlimb ischaemia models and MI models [102] (Fig. 4A).

Considering the possible risk of the direct use of native cell secretions containing various unknown factors, some verified effective elements from paracrine factors (such as certain miRNAs) and drugs were loaded into cell membranes to mediate the therapeutic effects. Yao et al. reported the self-assembly of MSC membrane-camouflaged miRNA-loaded mesoporous silica NPs. The MSC membrane helped NPs target ischaemic cardiomyocytes, where miRNA inhibited the translation of apoptosis-associated proteins. As a result, these NPs promoted the proliferation of cardiomyocytes and thus led to preservation of surviving myocardium and cardiac functions in the MI mouse model [103]. Zhang et al. injected MSC membrane-disguised Fe_3_O_4_ NPs into rats with articular cartilage defects, where the NPs encapsuling the cartilage regeneration-promoting drug kartogenin were able to more efficiently promote the regeneration of a layer of hyaline-like cartilage with an organized structure [104].

### Native cell membrane-based therapy without stem cell components

The abovementioned evidence indicates that the therapeutic effect in tissue repair could be achieved without stem cell membranes or paracrine signalling. Therefore, more combinations of various types of membranes from RBCs, PLTs or leukocytes and therapeutic cargos have been assessed.

In atherosclerosis models, either rapamycin‐loaded NPs with RBC or PLT membranes reduced macrophage‐mediated phagocytosis and enhanced plaque accumulation of NPs, which ultimately attenuated disease progression [109, 110]. Lumbrokinase was also used in thrombolysis with the targeting capacity of the PLT membrane [111]. Drug-loaded poly(lactic‐co‐glycolic) acid (PLGA) NPs could be targeted to sclerotic aortic valves through PLT membrane coating, which fully mimics the inherent adhesive interaction between PLT glycoproteins and components present in sclerotic aortic valves [112]. Without any cargos, the membrane could also be utilized in tissue repair. Li et al. fabricated PLT membrane-derived biomimetic nanovesicles for lesion accumulation to recanalize the obstructed vessels by inhibiting blood PLT aggregation in damaged blood vessels to protect the neural cells around the ischaemic area of the stroke, which was possibly mediated by integrin activation and angiogenesis-related receptors in PLTs [113].

Leucocyte membranes were subsequently used in cell membrane-based therapy. In addition to their inflammation targeting ability, leukocytes have shown the capacity to bind and neutralize proinflammatory cytokines, which could trigger immune activation and tissue injury. Zhang et al. integrated neutrophil membranes onto polymeric cores with the antigenic exterior and associated membrane functions of inherited neutrophils, which made them ideal decoys for biological molecules targeting neutrophils. The coating membrane could neutralize proinflammatory cytokines and thus suppress synovial inflammation and provide powerful chondroprotection against joint injuries [114]. By loading drugs or enzymes into neutrophil- or macrophage membrane-coated NPs, researchers could apply similar approaches to ameliorate inflammation in various tissue injuries, such as acute pancreatitis [115], ischaemia–reperfusion (I/R) injury [116], atherosclerosis [117] and gouty arthritis [118].

Recently, more types of cell membranes have been applied in NP coatings in addition to those of stem cells and haematocytes. To overcome the low drug accumulation in the substantia nigra and striatum, which is the main challenge of Parkinson disease (PD) treatment, Liu et al. camouflaged NPs with membranes from MES23.5, which is a hybrid cell line derived after fusion of rat embryonic mesencephalon cells with mouse neuroblastoma. Possessing the biofunctional surface of substantia nigra dopaminergic neurons, these membrane-coated NPs could effectively target microglia in the substantia nigra through interactions between their surface vascular cell adhering molecule 1 (VCAM1) and α4β1 integrin on microglia and provide NPs with the ability to modulate microglial overactivation for PD treatment [119]. A blood–brain barrier (BBB)-permeating capacity of some malignant tumours during metastasis has been reported. He et al. constructed NPs coated with membranes from the breast cancer cell line 4T1 to target cerebral ischaemic lesions, where ischaemia/reperfusion injury could be attenuated. CD138 and VCAM1 on 4T1 membranes have a high affinity for CD31 and very late antigen-4 on cells enriched in perivascular and inflammatory regions of the brain, which helps NPs migrate across the BBB to targeted cerebral ischaemic regions, achieving a 4.79-fold higher ischaemic hemisphere delivery efficiency compared to that of the normal side and a stronger ability to reduce the infarct volume than nude NPs [120].

Additionally, the targeting capacity of native cell membrane-coated NPs could be further enhanced by combining nanotechnology in cargo design. The inflammatory response in multiple tissue injuries could overproduce reactive oxygen species (ROS) [121–123]. Through self-assembly of amphiphilic oxidation-sensitive chitosan oligosaccharides, Gao et al. prepared ROS-responsive NPs that were degradable in the presence of abundant H_2_O_2_ in inflammatory tissues. By macrophage membrane coating, the ROS-responsive property of NPs enabled specific payload atorvastatin release after being led to inflammatory tissues, attenuating the proinflammatory efficacy of macrophages and the formation of foam cells in atherosclerosis [121]. A similar inflammatory regulating efficacy was also achieved in ulcerative colitis treated with an ROS-sensitive core loaded with rosiglitazone within the macrophage membrane [122]. Promoted targeting capacity could also be achieved under the navigation of an external magnetic field for cell membrane coating γ-Fe_2_O_3_ magnetic NPs [105] (Fig. 4B) or by membrane-coated chirally modified Pd catalysts and the asymmetric transfer hydrogenation reaction for selectively chiral drug synthesis in living cells at inflammation sites [124].

In addition to restricted systemic administration, the cell membrane could be utilized to improve scaffold function. There are two approaches to produce cell membrane-based scaffolds. For one approach, cell membranes are chemically incorporated in the scaffold that is already formed. Through the coating of cell membranes on drug-loaded PLGA, the problem of foreign body response to scaffolds could be eliminated, providing a strategy of anti-inflammatory protection for scaffolds [125]. For example, biological heart valves modified by glutaraldehyde are disadvantageous for cytotoxicity, the immune response, thrombus formation, difficulty with endothelialization and calcification, which limits their clinical application. Considering the large number of carboxyl groups as well as a few amino groups on the surface of these valves, Hu et al. crosslinked them with RBC membrane-camouflaged PLGA NPs loaded with rapamycin and atorvastatin. The modified heart valves preserved the properties of stability, structural integrity and mechanical properties and greatly improved anticoagulation and endothelialization and exerted anti-inflammatory and calcification effects after valve implantation [126].

In contrast to previous studies focusing on the development of spherical membrane-coated NPs, Chen et al. extended cell membrane coating technology to nanofibres, which are a type of material entirely different from NPs in dimensional and mechanochemical characteristics. Cell membrane-coated nanofibres were developed using pancreatic beta cells. When used to culture beta cells, the modified polymeric nanofibres significantly enhanced both cell function and proliferation rate, increasing glucose-dependent insulin secretion from the cells by nearly fivefold [127]. A similar approach was also applied to fabricate monocyte-camouflaged nanocellulose-coated polystyrene grafts for negative inflammatory signal transduction, suppressing the inflammatory responses of stimulated macrophages [128]. Tao et al. coated autologous rat RBC membranes on the surface of xenogeneic porcine extracellular matrix-based cartilage tissue grafts as a disguise. The cell membrane coating was highly stable without an obvious decrease in amount for 4 weeks and induced fewer inflammatory responses [106] (Fig. 4C).

In another approach, cell membranes are mixed with chemical materials to form a conjugate and then initiate the chemical crosslinking process for the formation of a hydrogel. For example, Fan et al. chemically crosslinked vesicles derived from RBC membranes with alginate, followed by crosslinking them with methacrylate to produce soft injectable scaffolds for efficient encapsulation and sustainable release of hydrophobic therapeutics. After subcutaneous injection in mice, the scaffolds showed low neutrophil infiltration and a higher number of infiltrated macrophages of the anti-inflammatory M2 phenotype [129].

### Engineering cell membrane-based therapy

Because membranes play a critical role in various cell functions, including adhesion, migration, and intercellular communication via surface proteins, numerous methods have been applied to enhance cell membrane function. One of the primitive approaches is to pretreat the original cells in stress-inducing conditions; for example, lipopolysaccharide-treated macrophage cell membranes have upregulated expression of cytokine receptors, which could prevent the inflammatory response once applied to coat NPs [130]. However, modifying the cell membrane with bioengineering approaches might be a more beneficial method to endow membrane‐camouflaged nanocarriers with properties other than those conferred by the native cell membrane [13]. The strategies for membrane engineering could be roughly classified as genetic and chemical approaches.

#### Genetic engineering of cell membranes

Proteins or peptides of interest could be introduced to cell membranes by genetic engineering through transfection or transduction via nonviral or viral vectors, respectively [131]. Among them, elements affecting targeting and therapeutic capacity are widely used to modify cell membranes. For example, membrane-harbouring CXCR4 could help achieve a higher level of nanocarrier accumulation in ischaemic tissue through its interaction with SDF-1, which is enriched in the ischaemic microenvironment. Through engineering of ASC membranes loaded with VEGFR to overexpress CXCR4 through transduction, combined with improved penetration ability across the endothelial cell barrier endowed by ASC membranes, these nanocarriers could substantially enhance blood reperfusion, muscle repair, and limb salvage in mice with hindlimb ischaemia [132]. A similar strategy of overexpressing CXCR4 on neural stem cell membranes could also significantly enhance the efficacy of stroke treatment with PLGA loaded with glyburide, an anti‐oedema agent [107] (Fig. 4D).

In addition to improved targeting capacity, genetic engineering could also improve the therapeutic effect of macrophage membranes to enhance cytokine binding with overexpressed interleukin‐1β (IL‐1β), interleukin‐6 (IL‐6), and tumour necrosis factor alpha (TNF‐α) receptors, which are induced by plasmid transfection. Combined with MI-protecting miR199a‐3p, these NPs could suppress inflammation, prevent hypoxia‐induced apoptosis, promote cell proliferation and inhibit cardiac fibrosis in mice with MI [133]. Similarly, the overexpression of Toll-like receptor 4 (TLR4) on the macrophage membrane neutralized endotoxin and inhibited the endotoxin-mediated TLR4/NF-κB signalling pathway to suppress overactivated Kupffer cells when hepatic I/R injury occurred after orthotopic liver transplantation. Therefore, TLR4-enriched membrane-coated PLGA NPs can improve the suppression of inflammatory factor secretion [134]. In addition to cytokine neutralization, NPs coated with umbilical vein endothelial cells overexpressing tumour necrosis factor-related apoptosis-inducing ligand (TRAIL) could target and induce M1 macrophage apoptosis in collagen-induced arthritic mice, with deeper penetration, higher accumulation and longer retention in inflamed joints, suppressing synovial inflammation [135].

#### Chemical engineering of cell membrane

Strategies for chemical modification are mainly based on lipid‐lipid interactions with the membrane and various conjugations to active sites on the membrane, such as primary amine and thiol residues of proteins and hydroxyl residues of polysaccharides on the cell membrane [136]. Specifically, the incorporation of prostaglandin E_2_ into the PLT membrane for NP coating could take advantage of the native MI‐homing capacity of the PLT membrane and the prostaglandin E_2_ receptors overexpressed in the microenvironment of cardiac I/R injury, allowing NPs to target delivery of therapeutic cargos to the injured heart [137]. The RBC cell membrane inserted with stroke homing peptide could enhance targeted payload transportation to the ischaemic area [123].

Furthermore, multistage targeting capacity could be achieved via various modifications. Han et al. developed membrane-coated solid lipid NPs with attachment of a triphenylphosphine (TPP) cation and a 29 amino acid peptide derived from RVG (RVG29) to the surface of the membrane. The decoration of RVG29 on the membrane surface endows the NPs with the ability to cross the BBB for specific targeting to neurons. TPP further led the NPs to mitochondria after entering neurons, where loaded elements had the most effective therapeutic effects on AD symptoms [138, 139].

Chemical engineering could also be used in therapeutic element loading. Wu et al. decorated macrophage membrane-coated NPs with an apolipoprotein A-I mimetic peptide to inhibit oxidized low-density lipoprotein uptake and promote cholesterol efflux in intimal foam cells, which exhibited excellent antiatherosclerotic effects [140]. Moreover, the multistage targeting capacity and therapeutic element loading could be integrated as well. Xu et al. developed PLT membrane-coated NPs conjugated with TAT-peptide-coupled recombinant tissue plasminogen activator (rtPA). When the connecting structure was cleaved under the concentration of thrombin from the environment, rtPA was triggered to be released for thrombolysis, while the in situ exposure to the TAT peptide subsequently enhanced the penetration of NPs across the BBB into the ischaemic region for site-specific delivery of the neuroprotectant-loaded core [108, 141] (Fig. 4E).

Under certain situations, therapeutic cargos might undermine the targeting function of the optimal coating membrane type. Chemical insertion of targeting molecules could help to mediate the corresponding function. For example, a PLT membrane was the best surface coating tool for thrombus targeting, contributed by the thrombus-homing property of PLTs. However, in the study of Zhao et al., tirofiban, as an antagonist of the PLT glycoprotein IIb/IIIa receptor, was selected for antithrombotic therapy, which might compromise the targeting capability of the PLT membrane. Therefore, the RBC membrane was functionalized with the fibrin-targeting peptide CREKA. As controlled release of tirofiban was achieved with conjugation to the nanocarrier core through an H_2_O_2_-cleavable phenylboronic ester linkage, RBC membrane-coated tirofiban exhibited site-specific antithrombotic effects after efficient accumulation at the injured carotid artery in a thrombosis mouse model [142].

#### Other types of engineering of cell membranes

**Fig. 5:**
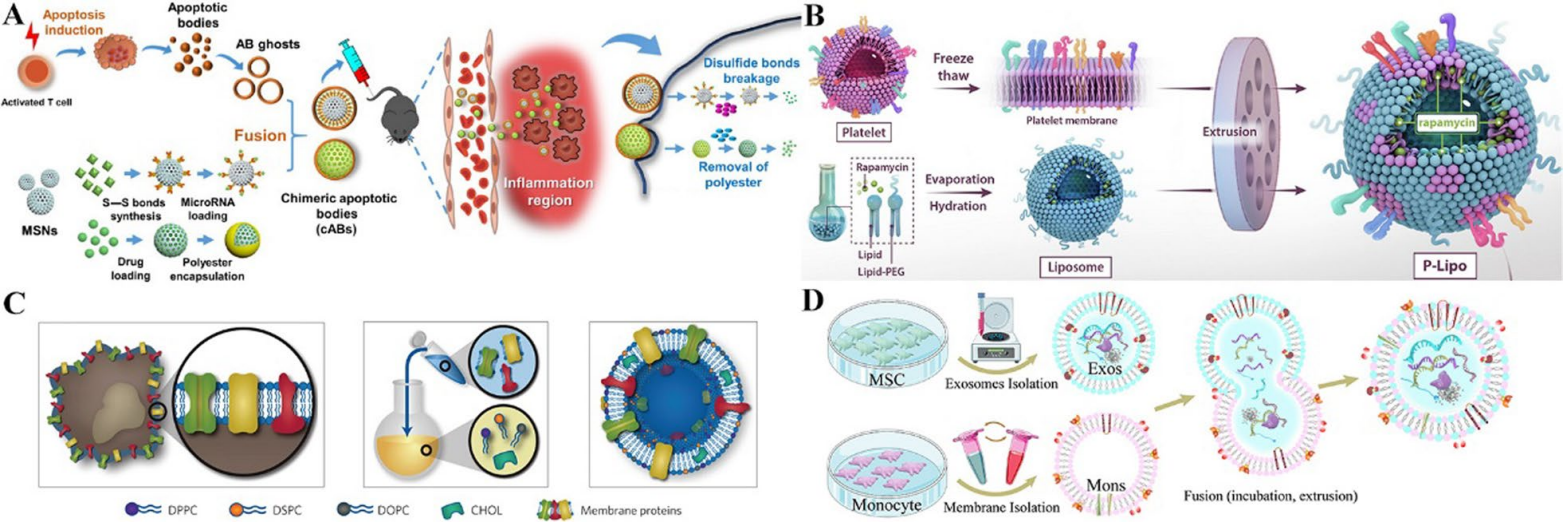
Other cell membrane-based therapy. **A** Schematic illustration of apoptotic bodies membrane coated mesoporous silica nanoparticle (MSN), which could actively target macrophages for inflammation modulation. Reproduced from [144]. © The Authors, some rights reserved; exclusive licensee AAAS. **B** Schematic illustration of liposome hybrids with platelet membranes for delivery of rapamycin targeting atherosclerosis. Reproduced from [147], copyright 2020, with permission from Elsevier. **C** Schematic illustration of cell membrane memetic therapy, which incorporated proteins derived from plasma membrane into lipid NPs to achieve both high surface complexity and better manufacture efficacy. Reproduced by permission from Springer [148], copyright 2016, https://doi.org/10.1038/nmat4644. **D** Schematic illustration of mesenchymal stem cell (MSC)-derived extracellular vesicles (EVs) fused with monocyte membranes for improved efficacy of endothelial maturation promotion and macrophage subpopulation modulation. Reproduced from [149], copyright 2020, with permission from Elsevier

Apoptotic cell efferocytosis could induce a phenotypic transition of macrophages towards proreparative M2 macrophages, facilitating the resolution of inflammation. NPs coated on apoptosis-featured phosphatidylserine (PS)-supplemented cell membranes could mimic crucial properties of the apoptotic cell surface, exerting anti-inflammatory effects by being engulfed by macrophages such as apoptotic cells, which could help mitigate macrophage-mediated inflammation [143]. Furthermore, Dou et al. directly constructed apoptotic body membrane-coated NPs for inflammatory modulation. Apoptotic body-like NPs were prepared to actively target macrophages, effectively promoting M2 polarization via the synergistic effects of apoptotic body membranes and intracellular cargo release [144] (Fig. 5A).

Although coated with the cell membrane, NPs might still induce an immune response in vivo after membrane camouflage is washed out. Therefore, researchers are also exploring methods to load therapeutic elements without NPs. Dong et al. directly loaded resolvin D2 (RvD2) into neutrophil membrane-derived nanovesicles, enhancing the resolution of inflammation, as RvD2 was able to incorporate into the lipid bilayer of nanovesicles by its lipid structure [145]. In another study, Wu et al. innovatively functionalized curcumin-loaded liposomes with stem cell membranes through ultrasonic cell rupture to avoid the use of NPs, generating controlled release in a low-pH environment, with good stability, long circulation time, and targeted delivery in a stroke model [146]. A similar strategy was applied in liposome hybrids with PLT membranes for atherosclerosis-targeting delivery of rapamycin [147] (Fig. 5B).

### Cell membrane mimetic therapy

Cell membrane-based therapy as a top-down approach has limitations due to the difficulties in controlling physical parameters such as size and homogeneity; controlling the packaging and reserve of chemically different molecules such as hydrophilic, lipophilic and amphiphilic molecules; and formulating a standardized protocol for manufacturing and preservation [150]. Molinaro et al. incorporated proteins derived from the macrophage plasma membrane into lipid NPs, combining bottom-up and top-down methods at the same time. Retaining the physicochemical and multifunctional characteristics typical of liposomal formulations, the resulting proteolipid nanovesicles could selectively target inflamed vasculature, enabling the preferential and efficient delivery of dexamethasone to inflamed tissues and preventing tissue damage by localizing to and alleviating inflammation. This approach endowed the NPs with a high surface complexity, which was achieved without chemical synthesis or complex purification steps. By taking advantage of the capacity of liposomes for loading, retaining, and releasing different payloads, this technique might lead to the elevation of yield and standardization of the manufacturing process for stable and nonimmunogenic products [148] (Fig. 5C). A similar approach was applied to develop biomimetic nanovesicles consisting of phospholipids, cholesterol and retinoic acid-induced membrane proteins from leukocytes with α4β7 integrin, which helped homing to inflamed tissue after its bond to receptors on the endothelial membrane [151]. Boada et al. recently reported this approach in the treatment of atherosclerosis [152].

As this bottom-up approach provides more tunability in the formulation design of NPs, the physicochemical properties and biomimetic behaviour of these NPs might change under variable biological conditions. For example, based on the assumption that the protein content on the NPs was related to their biomimetic targeting capacity, Zinger et al. constructed anti-inflammatory NPs with intact structural properties and improved inflamed endothelium-targeting capability by optimizing the protein-lipid ratio at a maximum of 1:20 (w/w) in the synthetic process [153].

### Combination of cell membrane-based therapy with cells or EVs

Cell membrane-based therapies do not always contradict cell therapies or EV therapies, and sometimes their combination could lead to unexpectedly excellent outcomes.

By combining cell membranes with cell therapies, Tang et al. showed that cardiac stem cells (CSCs) with PLT membranes fused onto their surface exhibited PLT surface markers associated with adhesion of PLTs to injury sites. The modified CSCs could thus preferentially bind collagen-coated and endothelium-denuded aortas in rat and porcine models of acute MI, enhancing their retention in the heart, where they could repair tissue and reduce infarct area. Through the conjugation of PLT membranes, this manipulation method for stem cells is convenient and safe, requiring no genetic editing of the cells, and might be applied to various other cell types [154]. Li et al. reported a PLT hybrid microglial platform. Through the fusion of PLT membranes on engineered microglia, these microglia were endowed with strong adherence to the injured cerebral vessels, where ultrasound-responsive IL-4 liposomes could promote on-demand M2 polarization of microglia, reducing apoptosis and promoting neurogenesis and long-term stroke recovery [155].

In terms of the incorporation of cell membrane-based therapies with EVs, Zhang et al. aimed at the obstacles of poor homing efficiency and low yields of stem cell-derived EVs and enhanced the targeted delivery capacity to ischaemia-injured myocardium of MSC-derived EVs with monocyte membranes through membrane fusion. By mimicking the recruitment feature of monocytes through exclusive adhesive molecules, particularly the interaction between intercellular adhesion molecule 1 and lymphocyte function-associated Ag-1 or macrophage-1 antigen, these EVs exhibited improved efficacy for endothelial maturation promotion and macrophage subpopulation modulation and improved therapeutic outcomes in cardiac function and pathohistological changes [149] (Fig. 5D). With another method, Lee et al. produced macrophage membrane-modified MSC-derived nanovesicles through extrusion of pregenerated macrophage membrane-camouflaged MSCs. These nanovesicles contained various receptors of inflammatory cytokines, such as integrin α4 and integrin β1, from macrophage membranes. These proteins facilitated the targeting of nanovesicles to ischaemic and inflammatory sites in the injured spinal cord, where they attenuated apoptosis and inflammation and enhanced blood vessel formation for the functional recovery of the spinal cord [156].

## Challenges for nonliving cell-derived therapy

Although nonliving cell-derived therapy, including EV therapy and cell membrane-based therapy, holds promise in tissue repair and regenerative medicine, this field is still in its early stages with multiple technical obstacles hindering its clinical translation.

### Challenges in therapeutic efficacy

Although multiple novel methods and materials have been used to establish advanced EVs and cell membrane-coated NPs, there are still doubts about their ability to improve the efficacy of nonliving cell-derived therapies. Most doubts are related to two key questions: I. EVs or cell membrane? II. Native or engineered?

For the selection of EVs or cell membranes before engineering, it is vital to clarify the main difference between them and their cargos. In contrast to the fact that cell membrane-based therapies are usually based on functional NPs such as cargos, EV therapies generally rely on endogenous cargos within EVs, with or without engineering. However, these EV cargos could lose therapeutic efficacy in the process of engineering; for example, EVs or exogenous cargos such as siRNA might aggregate during electroporation [93]. Additionally, exogenous reinforcement might overwhelm the better organized function of these complex natural therapeutic elements contained in EVs, which could ultimately compromise the efficacy of EV therapies [40]. Moreover, it is worth noting that the various bioactive factors in EVs might cause unpredictable side effects [46]. Therefore, in terms of question one, it seems that the cell membrane might be a more viable platform for cargo loading.

While the function of the EV membrane could be replaced by the plasma membrane in most situations (pristine EV membrane-coated NPs are more common in tumour homologous targeting), the main advantage of EV therapies is the organized function of complex EV cargos, which is irreplaceable by the combination of known factors or drugs. Once the question of how EVs actually exert their ability in a specific injury, namely, what specific EV functions are mediated by what specific cargos, is answered, the efficacy of endogenous EV cargos could be replaced [45, 157]. At that time, engineered cell membrane-coated NPs equipped with all desired therapeutic factors might be a better tool to address target injury. Before that, balancing the cons and pros of engineering is important. Genetic engineering of EVs and cell membranes by overexpressing a certain element in the source cell could lead to unanticipated biological impacts, which might ultimately interfere with EV biogenesis and the functions of EVs and cell membranes. Furthermore, genetic engineering might result in difficulties in controlling the density of therapeutic elements on EVs or the cell membrane. Although chemical engineering might allow effective control of both the structure and density of therapeutic elements during surface modification, it could also conceal or impair endogenous EV or cell membrane properties and ultimately compromise its bioactivity [40]. Therefore, it might be unnecessary to extensively attempt to engineer EV cargos, which answers the second question. Nevertheless, similar to EV cargos, membranes such as inflammatory cell membranes also show multiple neutralization functions for various inflammatory factors [150]. Thus, defining the extensive functions and effective factors of the cell membrane in targeting, immune evasion, neutralization and specific cellular communication might further contribute to the establishment of more engineered biomimetic machines.

Combining the answers to the first and second questions, a rule for the decision to choose native or engineered EVs or cell membranes could be acquired. Depending on different tissue injuries, from some that have been studied thoroughly to those whose mechanisms are unknown, diverse therapies could be applied. The better we know an injury, the more specific therapeutic elements and more engineered methods should be utilized to repair this kind of injury to enhance the known therapeutic mechanisms. For injuries that have not been thoroughly investigated, a more natural approach might be applicable before defined therapeutic or targeted elements are found to use for the repair of these injuries.

### Challenges in therapy translation

To date, no consensus on the optimal approach for EV isolation has been reached, as each isolation technique has its own advantages and limitations. Among various techniques, EV aggregation and changes in EV structure might be induced by differential ultracentrifugation, and there is a risk for coisolation of protein aggregates with other soluble factors. In size-exclusion chromatography, potential EV loss and increased processing times might be caused by the need for concentration of EV-containing medium before injection into purification columns [92]. Similar to EV isolation, there is no gold standard for cell membrane extraction and coating procedures. Typical protocols, including cell lysis, sonication, centrifugation, and extrusion, would cause loss in each step, and the uniformity of the membrane might be hard to control [150]. Additionally, it is important to maintain the original therapeutic cell phenotypes in large‐scale expansion. Changed phenotypes will make it difficult to control the purity and quality of the whole production [136].

For clinical translation, the EV dosage regimen in EV therapy has been highly contradictory, as a broad range of EV doses of 30–1300 μg/kg and 4–4000 μg/kg have been applied to vascular disease in rats and mice, respectively [40]. In humans, a clinical study has shown that 10^10^–10^11^ EVs were an effective therapeutic dosage to treat one patient with graft-versus-host disease [158], while other studies suggest that > 10^14^ vesicles per dose might be required for the clinical application of EVs [159]. As a more engineered approach, cell membrane-based techniques might have fewer dosage problems. However, it needs to be noted that the use of different kinds of EVs or membranes might largely depend on the concomitant disease in the patient and their medication. For example, patients taking anti-PLT drugs might not be suitable for PLT EV or membrane-based therapy.

As a result, large-scale and standardized experiments are needed to compare these various approaches for the corresponding cell-derived biomaterials and to formulate the general dosage regimen. However, malignant cells might help in the large-scale manufacture of nonliving cell-derived therapy. Research has shown that a malignant T cell line (EL4 cells) rather than primary T cells could be used in the cell membrane coating technique, with a low risk of tumorigenesis in vivo due to elimination of tumorigenic genetic materials, which is cost‐effective and less time‐consuming than adoptive T cell transfer therapy [160]. The utilization of these kinds of malignant cells might solve the problem of the shortage of some source cells that are hard to obtain or expand. Large-scale cell production by programming PSCs to desired cells [161] and cell membrane mimicking by incorporating defined membrane proteins into the lipid NPs mentioned above [148] might also be beneficial for large-scale manufacturing. In terms of controlling cell phenotypic changes during the production process, 3D bioreactors might play an important role in large‐scale culture with better lineage control [102]. However, considering the potential immune rejection reaction, convenient methods for the large‐scale manufacture of autologous cells are still urgently needed.

## Conclusion and outlook

The development from cell therapy to EV therapy and cell membrane-based therapy for tissue repair is at the same time the evolution from a natural approach to an engineered approach with the mechanism of cell therapy efficacy fulfilled by paracrine factors being clear. In this review, we systemically summarize the utilization of multiple native or engineered EVs and cell membranes combining advanced bioengineering techniques in the treatment of various tissue injuries. Although there are still challenges for functional realization and clinical translation, the accumulation of evidence suggests that these nonliving cell-derived therapies, especially cell membrane-based therapies, have great potential in the field of tissue repair due to their strong ability to combine with novel advanced materials and methods. However, as a newcomer in the regenerative field, this kind of technique has a much greater potential than those applications mentioned above. There are multiple new fields, advanced coating techniques and biomimetic working mechanisms of cell membrane-based therapy in tissue repair and regeneration. By combining cell membrane-coating technology and advanced materials, researchers can address a variety of problems in NP applications. For example, the use of NPs usually results in endothelial cell leakiness, which is caused by interactions with the vasculature, including physical interactions between NPs and endothelial cell adherens junction proteins [162, 163]. Leukocytes, including neutrophils and monocytes, can migrate through vascular walls to sites of injury by adhesiveness between membranes and shape changes under hydrodynamic forces [164]. Moreover, deformable particles such as RBC-mimic particles designed by Hayashi et al. [165] could pass through pores smaller than their diameter by virtue of intraparticle elasticity distribution. Through application of the leukocyte membranes mentioned previously to camouflage these flexible materials, the endothelial cell leakiness induced by NPs might be promisingly prevented.

In regard to the origin of the coating membrane, Gong et al. recently performed pioneering work to introduce intracellular membranes of organelles to NP camouflage. In their research, the outer mitochondrial membrane (OMM) from mouse livers was coated onto NPs as a representative. By protecting cells from cell death and apoptosis induced by the B-cell lymphoma protein 2 inhibitor ABT-263, which targets the OMM, OMM-coated NPs could ameliorate thrombocytopenia, which is a major adverse effect in the clinical use of ABT-263 [166]. In tissue injuries, various inflammatory stresses could induce mitochondrial outer membrane permeabilization, which would result in the release of damage-associated molecular patterns such as cytochrome C and mtDNA into the cytosol, activating further inflammatory injuries [167, 168]. With the help of OMM-coated NPs as sponges, these mitochondria-related injuries could be ameliorated. Such an advanced technique allows the coating of other intracellular membranes, such as the nucleus, endoplasmic reticulum, Golgi apparatus, and lysosomes, for novel biointerfacing applications, as the same basic structure is shared between them and mitochondria. Moreover, multiple nonlipid biomimetic NPs have recently been developed, providing a platform for design strategies that are more bottom-up for functional realization. For example, Mukwaya et al. developed an NP system for cognate signaling cascades based on the modification of surface-attached synthetic virus-like particles on a polysaccharide-polymer semipermeable membrane, which was an alternative to biological membranes and had higher permeability than lipid biomimetic entities such as liposomes [169]. For further analysis of cell-mimetic NPs, both lipid [170] and nonlipid [171] elements have been utilized in multilayered cell-like membrane fabrication. The multilayered membranes enable the generation of artificial cells with compartments loading various therapeutic molecules, which might bring new competition between top-down and bottom-up NP design strategies [172].

In-depth understanding of the mechanism for tissue repair sometimes is far more matter than innovation in engineering. Defining new targets and cells for biomimetic engineering could be meaningful via research on the microenvironment of the injured area, where various cells continuously initiate communications, and different cells might exert multiple functions with the injury response developing both in time and space. For instance, with in-depth research on the glucose-responsive insulin secretion function of beta cells, Chen et al. constructed artificial beta cells equipped with a glucose metabolism and membrane fusion system possessing a multicompartmental 'vesicle-in-vesicle' superstructure, which had the function of beta cells and showed promise for improving clinical outcomes in people with diabetes [173]. Such a spontaneously responsive drug release system was established through an effort to thoroughly study the mechanism of the physiological process in the human body, which indicates that there is still a vast space for future research and development in the field of cell-derived biomaterials for tissue repair.

## Data Availability

The data supporting the conclusions of this article are included within the article.

## Abbreviations

EVs: Extracellular vesicles
ESCs: Embryonic stem cells
iPSCs: Induced pluripotent stem cells
HSCs: Hematopoietic stem cells
MSCs: Mesenchymal stem cells
PSCs: Pluripotent stem cells
hESCs: Human embryonic stem cells
MI: Myocardial infarction
BM-MSCs: Bone marrow–derived mesenchymal stem cells
HGF-eMSCs: Hepatocyte growth factor–expressing MSCs
IFN-γ: Interferon-gamma
Tregs: Regulatory T cells
hucMSC: Human umbilical cord mesenchymal stem cell
ASCs: Adipose-derived stem cells
RVG: Rabies viral glycoprotein
DC: Dendritic cell
IONP: Iron oxide nanoparticle
SEVs: Small EVs
AD: Alzheimer disease
RBCs: Red blood cells
PLT: Platelet
MPs: Microparticles
ASSP: Artificial stem cell spheroid
PLGA: Poly(lactic‐co‐glycolic) acid
I/R: Ischemia–reperfusion
PD: Parkinson disease
VCAM1: Vascular cells adhering molecule 1
BBB: Blood–brain barrier
ROS: Reactive oxygen species
IL: Interleukin
TNF‐α: Tumour necrosis factor alpha
TLR4: Toll-like receptor 4
TRAIL: Tumour necrosis factor-related apoptosis-inducing ligand
TPP: Triphenylphosphine
rtPA: Recombinant tissue plasminogen activator
PS: Phosphatidylserine
RvD2: Resolvin D2
MSN: Mesoporous silica nanoparticle
CSCs: Cardiac stem cells
OMM: Outer mitochondrial membrane
